# Flourishing tumor organoids: History, emerging technology, and application

**DOI:** 10.1002/btm2.10559

**Published:** 2023-06-07

**Authors:** Qian Yang, Mengmeng Li, Xinming Yang, Zian Xiao, Xinying Tong, Ayinuer Tuerdi, Shisheng Li, Lanjie Lei

**Affiliations:** ^1^ Department of Otorhinolaryngology Head and Neck Surgery, the Second Xiangya Hospital Central South University Changsha Hunan China; ^2^ Department of Hemodialysis, the Second Xiangya Hospital Central South University Changsha Hunan China; ^3^ State Key Laboratory of Bioelectronics, School of Biological Science and Medical Engineering Southeast University Nanjing China

**Keywords:** CRISPR‐Cas9, drug screening, precision medicine, three‐dimensional culture, tumor organoid

## Abstract

Malignant tumors are one of the leading causes of death which impose an increasingly heavy burden on all countries. Therefore, the establishment of research models that closely resemble original tumor characteristics is crucial to further understanding the mechanisms of malignant tumor development, developing safer and more effective drugs, and formulating personalized treatment plans. Recently, organoids have been widely used in tumor research owing to their advantages including preserving the structure, heterogeneity, and cellular functions of the original tumor, together with the ease of manipulation. This review describes the history and characteristics of tumor organoids and the synergistic combination of three‐dimensional (3D) culture approaches for tumor organoids with emerging technologies, including tissue‐engineered cell scaffolds, microfluidic devices, 3D bioprinting, rotating wall vessels, and clustered regularly interspaced short palindromic repeats‐CRISPR‐associated protein 9 (CRISPR‐Cas9). Additionally, the progress in research and the applications in basic and clinical research of tumor organoid models are summarized. This includes studies of the mechanism of tumor development, drug development and screening, precision medicine, immunotherapy, and simulation of the tumor microenvironment. Finally, the existing shortcomings of tumor organoids and possible future directions are discussed.

## 1 INTRODUCTION

Malignant tumors are one of the leading causes of death which impose a significant burden on individual countries worldwide. This burden will gradually increase over time, with the global cancer burden predicted to reach 28.4 million cases by 2040.^1^ Despite some achievements in the diagnosis and treatment of malignant tumors, several challenges remain.^2^ Little is known about the mechanisms involved in tumor development, progression, and the development of drug resistance. Different individuals with the same tumor may respond differently to treatment because of the complexity of tumors. Therefore, establishing models that closely resemble in vivo tumors will improve our understanding of malignant tumors and help formulate more effective treatment plans.

Currently, preclinical models applied in tumor research mainly include two‐dimensional (2D) cultured cell lines, patient‐derived tumor xenografts (PDXs), and patient‐derived tumor organoids (PDOs).^3^ 2D cultured cell lines have been used for the longest period of time compared to the other two models, and the methods for establishing this model simpler and easier to use for tumor research and drug screening. However, the composition of tumors is more complex, and cell line cultures are usually derived from a single cell that does not mimic the real state of tumor cells in vivo and their interaction with the in vivo environment, which may lead to a misestimation of the model's response to therapy in vivo.^4^, ^5^ The patient‐derived tumor xenograft is a preclinical model wherein patient‐derived tissue is transplanted into immunodeficient mice; this preserves the heterogeneity of the tumor and tumor–stroma interactions^6^, ^7^ where orthotopic transplantation is closer to the real environment than subcutaneous transplantation.^8^ However, the tumor microenvironment (TME) transplanted into mice is gradually being replaced by murine‐derived cells.^9^ Tumor cells exhibit different evolutionary trajectories in patients and mouse models^10^ and the lack of a normal immune microenvironment makes PDXs‐based results potentially deviant from the real situation.^11^, ^12^ Moreover, drawbacks such as the complexity of the operation, low success rate, and unsuitability for gene editing limit the application of PDXs. Patient‐derived tumor organoids are three‐dimensional (3D) cultures of tumor tissue from patients that can preserve the histological and genetic characteristics and heterogeneity of the original tumor under in vitro culture conditions.^13^ This method overcomes some of the drawbacks of 2D cultured cell lines and PDXs and has great potential for high‐throughput drug screening and personalized medicine.^14^

In this review, we introduce the history and characteristics of tumor organoids, and analyze the synergistic combination of 3D culture methods for tumor organoids with emerging technologies, including tissue engineering cell scaffolds, microfluidic devices, 3D bioprinting, rotating wall vessels (RWVs), and clustered regularly interspaced short palindromic repeats‐CRISPR‐associated protein 9 (CRISPR‐Cas9). In addition, the research progress of tumor organoid models and their applications in basic and clinical research are discussed, including studies involving the mechanisms of tumorigenesis and tumor development, drug development and screening, precision medicine, immunotherapy, and simulation of the TME. Finally, the existing shortcomings of tumor organoids and scope for the future are discussed.

## 2 HISTORY OF TUMOR ORGANOIDS

**FIGURE 1:**
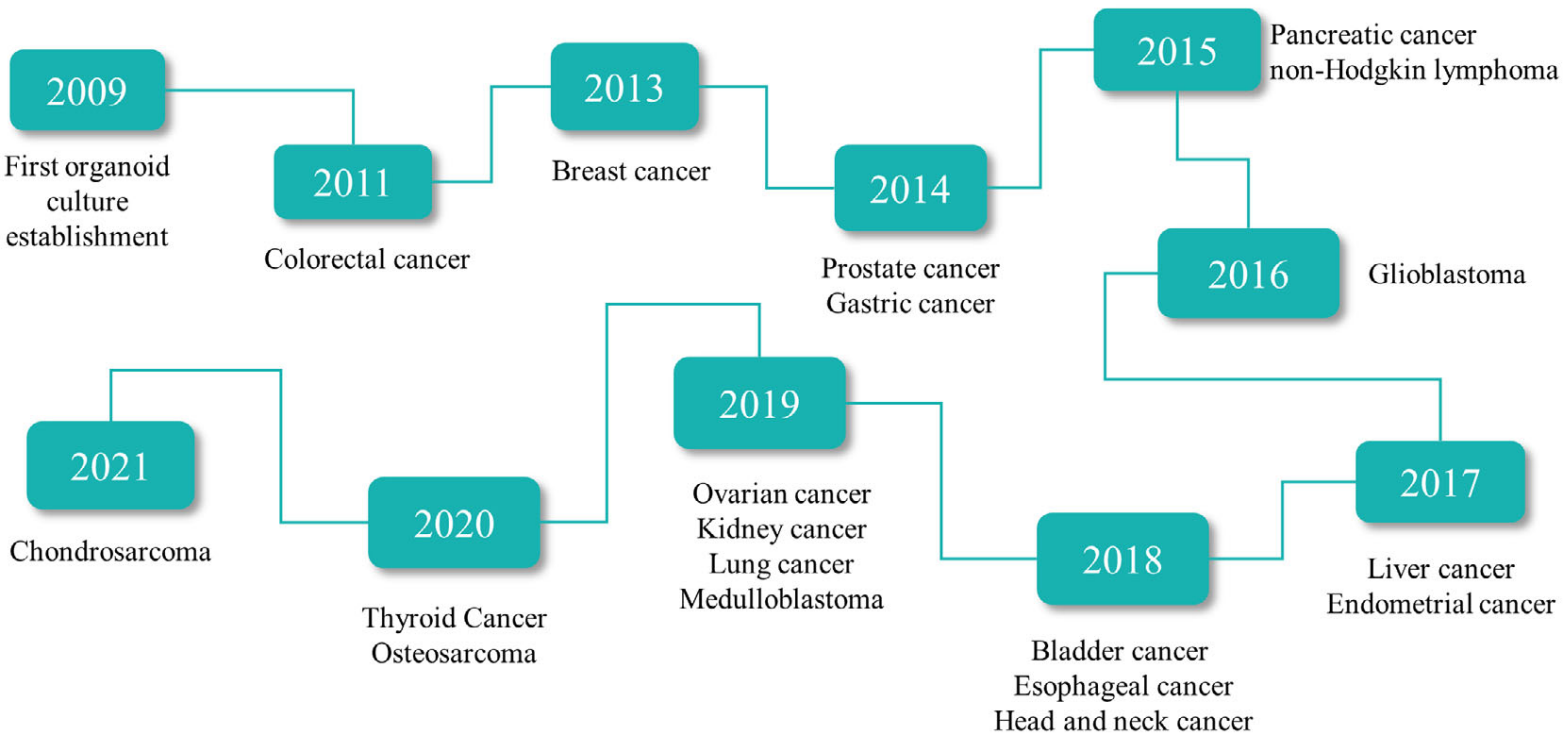
The timeline of tumor organoids development. Different organoid protocols for human tumors have been reported for the first time.

Currently, the establishment of organoids is based on the ability of cells to self‐organize and differentiate, and form in vitro 3D culture. In 1907, Henry Van Peters Wilson demonstrated that isolated sponge cells can form regenerative tissues through self‐organization.^15^ Subsequently, studies on the in vitro culture of animal organs slowly developed in the laboratory.^16^, ^17^ In 1981, pluripotent stem cells (PSC) were isolated from early mouse embryos cultured in a medium conditioned by teratocarcinoma stem cells.^18^ Eighteen years later, ESCs were first extracted from human blastocysts.^19^ The development of organoid research is increasingly flourishing as scientists continue to understand and study stem cells and reduce the use of animal models. In 1987, Li et al.^20^ found that mouse mammary epithelial cells cultured on recombinant basement membranes from Engelbreth–Holm–Swarm tumors (EHS) form ducts and lumens. They exhibit a structure different from previous in vitro cell cultures and provide an idea for the development of 3D cell culture. In 2008, Eiraku et al. induced the differentiation of ESCs into self‐organized, functionally polarized cortical tissues using 3D aggregation culture.^21^ This led to a shift in the study of organoids from 2D to 3D formats. Subsequently, Sato et al. successfully established 3D mouse crypt fossa structures in a new matrix gel culture system and maintained them for 8 months.^22^ This was a landmark discovery in the history of organoid development in 2009, and almost all other tissue‐derived organoids are established based on this study. Since then, research on organoids has rapidly developed. To date, successfully established human organoids include the colon,^23^, ^24^ stomach,^25^, ^26^ liver,^27^, ^28^, ^29^ pancreas,^29^, ^30^ lung,^31^, ^32^, ^33^ and kidney.^34^, ^35^ The establishment of organoids can reduce the number of experimental animals to some extent. Also, they can reduce some of the experimental errors caused by species differences as they are based on human genetic backgrounds. Meanwhile, the establishment of PDOs has been increasingly improving owing to the demand for tumor research, including colon,^23^, ^36^, ^37^ gastric,^38^ prostate,^39^, ^40^ pancreatic,^41^, ^42^ breast,^43^, ^44^ bladder,^45^ and esophageal cancers^46^ (Figure 1). Currently, PDOs are also widely used because of their similar histological, genetic, and cellular behavioral characteristics as those of parental tumors.

## 3 METHODS FOR CULTURING TUMOR ORGANOIDS

### 3.1 Traditional culture methods

The culture protocol for PDOs usually includes an ECM substitute (Matrigel or basement membrane extract), growth factors, molecule inhibitors, and a culture medium.^3^ In general, the culture of tumor organoids follows the following procedure. First, the obtained tumor tissue is processed into small pieces or cells by mechanical methods and enzymatic digestion, then inoculated into the ECM substitute and cultured in a tissue‐specific medium.^14^ Growth factors and ECM substitutes can be involved in the regulation of cell signaling which affects cell behavior.^47^, ^48^ For example, primary breast cancer epithelial cells of the same origin do not behave similarly when cultured in normal and abnormal ECM‐mimicking environments. In the normal ECM‐simulated environment, the tumor cells showed inert growth or aggregated growth. However, in the abnormal ECM‐simulated environment, the tumor cells showed protrusive migration and dissemination. In addition, removing the Wingless‐related integration site (Wnt) during organoid culture of colon cancer also achieved selective culture of cancer cells.^36^ A mixture of different tumor tissue‐specific growth factors can mimic paracrine signaling.^14^ Therefore, there is a slight variation in the composition of the medium and growth factors used for the organoid depending on the cell source and purpose of the study. Sometimes, a certain class of the component is necessary for one PDOs but not for another. For example, testosterone is required in prostate tumor organoids, but not in other tumor organoids.^49^ The culture route is also important for the generation of tumor organoids. Commonly used methods include submerged culture for organoids of gastrointestinal (GI) origin and air–liquid interface for organoids of respiratory and renal origin. However, larger organoids cannot be cultured using these two methods owing to the limitation of nutrient accessibility.^50^

Owing to a lack of vasculature, general organoids need to control their size during culturing. When organoids are large, cells near the center have difficulty exchanging oxygen and nutrients with the outer environment. Thus, the larger the structure size, the more number of cells die. Therefore, for organoids of normal cell or tissue origin, the maximum size of their culture does not exceed 4mm.^51^ For PDO, the size of organoids usually needs to be controlled below 100 μm in order to reduce central necrosis and meet the requirements of high throughput.^52^

The traditional culture protocol plays an extremely important role in the establishment and application of tumor organoids; however, this protocol has some drawbacks. For example, the commonly used ECM substitute (Matrigel) is derived from animals, but its composition is unclear, and its effect on the organoid grown in it is unknown.^53^ Second, samples are mainly derived from a limited sample source: surgically resected/biopsied tissues of patients. Furthermore, most tumor organoids only contain tumor cells and lack TME components such as stromal cells.^54^ Therefore, researchers have explored ways to improve existing ECM alternatives and to find new and more suitable ECM alternatives.

### 3.2 Emerging technologies

#### 3.2.1 Tissue‐engineered cell scaffolds

Matrigel is an indispensable component of many PDOs culture protocols. It is derived from Engelbreth–Holm–Swarm mouse sarcoma and its composition is essentially identical to that of the tumor ECM,^55^ including laminin (major component), collagen IV, heparin sulfate proteoglycan perlecan, entactin, and various growth factors.^56^ Considering its origin, Matrigel may increase the risk of animal‐derived pathogenic microbial infections and inter‐ and intra‐lot variability in organoids. This can lead to uncertain and unreproducible experimental results.^57^, ^58^, ^59^ These limitations have prompted researchers to search for and develop suitable ECM alternatives (Table 1).

**TABLE 1:** Different types of materials used in organoid culture.

Biomaterial	Material type	Features	Limitations	Reference
Matrigel	Natural	Cheap Easy access Wide application	Risk of animal‐derived pathogenic microbial infections Inter‐ and intra‐lot variability Poor control of mechanical properties	59
Natural biological macromolecules (e.g., collagen)	Natural	Good histocompatibility Easy access Biodegradability	Poor mechanical performance Biological and physical properties cannot be controlled independently Inter‐ and intra‐lot variability	70, 71
Synthetic polymer	Synthetic	Controllable mechanical properties Repeatability Adjustable degradation rate	Cell adherence only after modification Potential cytotoxicity Low similarity to natural tissue ECM	71, 75
Acellular ECM	Natural	Retains natural chemical and mechanical properties	Acquisition difficulty Unclear composition	80
Hybrid hydrogels (combination of two or more materials)	Synthetic	Suitable mechanical properties Low immunogenicity in vivo ECM‐like biology	Research still in infancy	236

Abbreviation: ECM, extracellular matrix.

In recent years, tissue‐engineered cell scaffolds provide support for cell growth and attachment and mimic the function of ECM in vivo with similar or superior results to Matrigel.^60^ Hydrogels are highly promising 3D scaffolds for tissue engineering applications because of their high‐water content, porous structure, and uniform cell‐loading capacity.^61^ Hydrogel scaffolds are composed of natural materials, synthetic hydrophilic polymers, or a mixture of both, with a water content of up to 95%,^62^ and good histocompatibility and permeability.^63^, ^64^, ^65^ Scaffolds with different compositions, molecular weights, and fabrication methods behave differently in terms of biochemical and biophysical aspects such as porosity, solubility, and compliance.^60^, ^66^, ^67^ This can have an impact on cell phenotype and behavior; therefore, it is essential to select a suitable ECM substitute according to the experimental specifications. Scaffolds of natural origin can be composed of one or more components including proteins (such as collagen) and polysaccharides (alginate or alginate–chitosan mixtures, hyaluronic acid, and mixtures of hyaluronic acid and chitosan).^68^, ^69^ Enzymatically crosslinked hydrogels are comparable to Matrigel for colorectal tumor organoid culture; this preserves the sensitivity of colorectal tumors to various therapeutic agents.^70^ Collagen or hyaluronic acid are natural components of ECM so they have good histocompatibility; however, their application is limited since they have poor mechanical properties, and their biological and physical properties cannot be independently controlled.^71^ In addition, the impact of collagen on tumor cells varies depending on its cellular origin, resulting in distinct biological manifestations of the corresponding PDO. For instance, pancreatic cancer cells specifically produce type I collagen (Col1) homotrimer (α1/α1/α1). This homotrimer promotes pancreatic cancer cell proliferation and tumor progression, while exhibiting tumor immunity and microbiome oncogenicity in mice. Compared to pancreatic cancer PDO without Col1 knockout, pancreatic cancer PDO with Col1 knockout had more retarded growth and development. In contrast, the normal Col1 heterotrimer (α1/α2/α1) produced by fibroblasts in pancreatic ductal adenocarcinoma (PDAC) exhibited immunosuppression and promotion of tumor progression after knockdown.^72^

Materials for synthetic scaffolds mainly include metals, ceramics, and organic or inorganic polymers such as polycaprolactone (PCL), polyacrylamide (PAM), and polyethylene glycol (PEG).^73^ The cell adhesion of the resulting scaffold made from polymer alone is poor; therefore, additional modifications are required to enable cell adhesion.^74^ Culturing of cancer‐associated fibroblasts (CAFs) on a PCL scaffold allows the deposition of CAF‐derived ECM on the scaffold; this promotes cell adhesion and growth through integrin‐mediated cell attachment.^75^ The mechanical environment and ECM components required at different stages of organoid generation vary. The ability to control the physicochemical properties of synthetic scaffolds extends their applicability to basic and clinical research.^76^

Recently, Below et al.^77^ described a well‐defined, fully synthetic hydrogel scaffold based on 8‐arm PEG and successfully established pancreatic cancer organoids to achieve organoid growth and polarization. Furthermore, pancreatic cancer organoid growth was modulated by altering the hydrogel properties. Furthermore, when co‐cultured with the stroma, organoid can bind to the stromal cells in the hydrogel and exhibit signaling, phenotypic, and tumor behavior consistent with the in vivo model. Synthetic scaffolds have a well‐defined and controlled composition with insignificant batch‐to‐batch variation, and their use in combination with well‐defined media can largely avoid the possibility of unknown proteins and genes affecting cell culture. However, the biocompatibility and cytotoxicity of synthetic scaffolds must also be considered.^78^, ^79^ Preparation of a GI tissue‐derived ECM hydrogel using an optimized decellularization scheme is rich in GI tissue‐specific core matrisomes and nonmatricellular proteins; this is an effective alternative to Matrigel.^80^ This allows for better support of the growth and development of gastrointestinal tract organoids and allows long‐term subculture and passaging of gastrointestinal tract organoids to generate more organoids. In addition to gastrointestinal sources, ECM hydrogel has now expanded to animal sources such as heart, bladder, kidney, lung, fat, tendon, and skeletal muscle, with the most prominent animal donor being the pig.^81^ However, the ECM in this hydrogel is derived from animals; therefore, it has some similar limitations to that of the Matrigel. Unlike animal‐derived ECM hydrogel, human‐derived ECM hydrogel is well protected from differences in ECM composition, xenogeneic reactions, and transmission of animal‐derived pathogens.^82^ Human‐derived ECM hydrogel can be derived from a variety of tissues/organ, such as skin, cartilage, tendon, adipose tissue, heart, lung, gastrointestinal tract, liver, pancreas‐kidney, gonads, uterus, umbilical cord, cornea, and peripheral nerves.^83^ Both animal‐derived and human‐derived ECM hydrogel have been commercialized for clinical applications, such as In Matrico® (porcine or human‐derived), GraftJacket® (human dermis), and Prima™ Plus (porcine heart valves).

#### 3.2.2 Microfluidic cell culture platforms and organ‐on‐a‐chip

**FIGURE 2:**
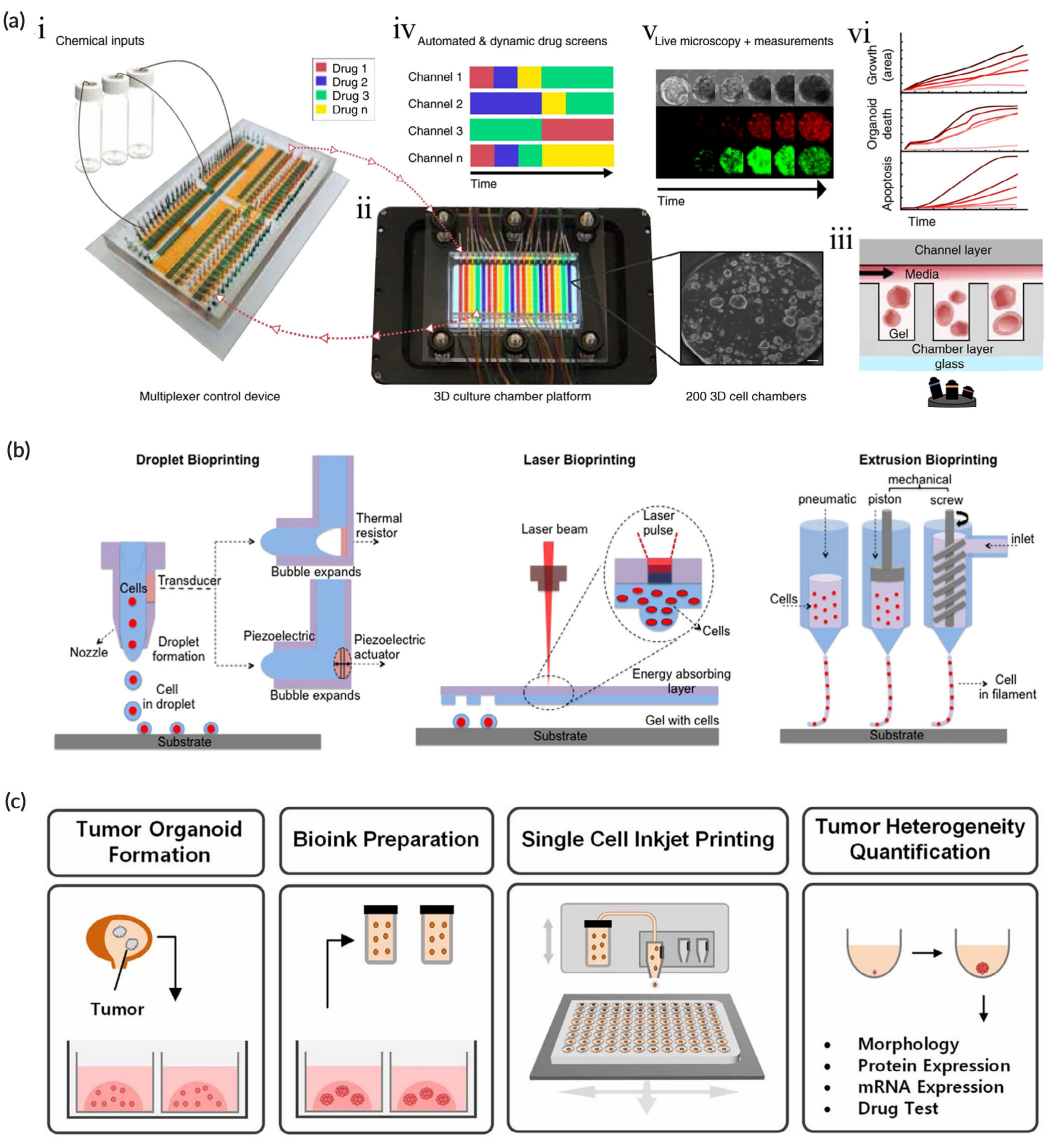
Tumor organoid culture based on microfluidic devices and tumor organoids based on 3D bioprinting. (a) Automated microfluidic 3D cellular and organoid culture platform for dynamical drug perturbations. (i) A programmable membrane valve‐based microfluidic chip. (ii) 3D culture chamber platform(scale bar 100 μm). (iii) Cross‐section of a pdms‐based bilayer multi‐chamber 3D culture chamber device(scale bar 100 μm). (iv) Automated and dynamic drug screens. (v) Continuous observation of organoid or 3D cellular structures by time‐lapse imaging. (vi) Quantification of organoid or 3D cellular structures. (Reproduced with permission from Reference 85. Copyright 2020, Springer Nature). (b) Schematic diagram of 3D bioprinting approaches. (Reproduced with permission from Reference 100. Copyright 2016, IOP Publishing). (c) Inkjet printing to quantify tumor heterogeneity. (Reproduced with permission from Reference 120. Copyright 2020, IOP Publishing).

Microfluidic devices are mainly used to simulate physiological and pathological parameters in a controlled 2D cell culture environment for drug screening.^84^ In recent years, progress has been made in the application of microfluidics in 3D cell culture. Schuster et al.^85^ described a microfluidic organoid culture system that is compatible with gel systems and allows automation and high throughput (Figure 2a). This culture system allows real‐time and reproducible analysis of organoids, and permits different combinations and temporal sequences to test the effects of drug treatments. Currently, microfluidic‐based organoid culture platforms (also referred to as organ‐on‐a‐chip or organoid‐on‐a‐chip) are reported for lung cancer,^86^ gastrointestinal tumors,^87^ and pancreatic cancer.^85^, ^88^ Microfluidic techniques can mimic the TME for tumor organoids, including blood supply,^89^ interaction with the immune system,^90^ and stromal cells.^91^ Shirure et al.^89^ established a microfluidic platform that mimics biological mass transport near the end of capillary arteries in a TME. This platform allows dynamic observation of the hallmark features of tumor progression (including cell proliferation, angiogenesis, cell migration, and intra‐tumor cell invasion) and induces sprouting angiogenesis. Microfluidics are more representative of the real in vivo tumor environment compared with tumor organoids alone, compensating for the lack of TME in tumor organoids, and are promising models for preclinical studies.^89^, ^92^, ^93^ However, organ‐on‐a‐chip models are currently unsuitable for widespread use because most are disposable devices and consume considerable time, money, and labor.^94^

Microfluidics can be used to study ECM surrogates. The dependence of breast cancer organoids on Matrigels has limited their application as preclinical models to some extent. Meanwhile, breast cancer organoids cultured in alginate microbeads using microfluidic droplet technology have similar structural and phenotypic properties to parental tumors while maintaining drug sensitivity.^95^ This suggests that alginate microbeads are potentially suitable for breast cancer organoid culture.

In conclusion, the combination of microfluidics and organoid culture helps to achieve a high‐throughput generation of organoids and improves the applicability of organoids in drug screening and personalized therapeutic applications. However, many of the combined applications of microfluidics and organoids reported in the literature have complicated processes, high costs, and the inability to accommodate large‐sized organoids. In addition, the following limitations exist in achieving high‐throughput and automated screening of PDO: (1) as the size of the assay is scaled up, the volume and number of cells of the required organoids change, for example, during the conversion from screening using 384‐well plates to screening using 1536‐well plates, the size of the organoids is not simply reduced, but further testing and optimization have to be performed before screening can be performed^96^; (2) high‐throughput screening saves material to some extent for organoid culture, but with it comes an increase in dead volume, which requires the purchase of additional equipment to address^97^; (3) as the number of wells per plate increases, the smaller the volume available, the impact that edge effects can have during culture can be significant.^98^

#### 3.2.3 3D bioprinting

3D bioprinting has rapidly developed in the last decade, with notable applications in regenerative medicine and tissue engineering.^99^ Bioprinting is the use of cells and/or other biocompatible materials such as bioinks for printing entities with 3D structures, including organs or tissues. The structure is formed layer by layer with precise control of the spatial and temporal distribution of cells and ECM at micron‐level resolution (Figure 2b).^100^, ^101^ There are three main bioprinting techniques available: droplet bioprinting,^102^ extrusion bioprinting,^103^ and laser bioprinting.^104^ Moreover, new bioprinting techniques include freeform reversible embedding of suspended hydrogels (FRESH),^105^, ^106^ acoustic,^107^ stereolithography,^108^ and magnetic.^109^ Droplet bioprinting was one of the first bioprinting methods used^110^ and involves a layer‐by‐layer printing of cells and inactive materials on a substrate via droplet encapsulation.^111^ Droplet bioprinting has the advantages of low cost, high speed (10 kHz), and high cell survival after printing; however, the inability to use high‐viscosity materials leads to poor mechanical properties of the printed structures.^100^, ^112^ Unlike droplet bioprinting, extrusion bioprinting can print structures with high cell density using high‐viscosity materials and maintains a faster speed and fair cell viability; however, it has low spatial resolution.^113^ Laser bioprinting is based on laser‐induced forward transfer and its post‐printing cell viability is higher than that of droplet bioprinting. Laser bioprinting allows for single‐cell control and does not suffer from clogging and high temperatures which are disadvantages of droplet and extrusion bioprinting; however, the high cost prevents the printing of large entities.^100^, ^111^ 3D bioprinting of skin,^114^ heart,^115^ bone,^116^ liver,^117^ and brain‐like tissue^118^ is reported, but complete organs that can be used for transplantation are not possible so far.

The combination of 3D bioprinting and tumor organoids is primarily used to develop high‐throughput PDOs models suitable for drug screening (Figure 2c).^119^, ^120^ Reid et al.^121^ described a method to place stem cells directly into a hydrogel using a low‐cost 3D printer that retains function and viability of stem cells for 7 days. The authors used the method to print breast cancer cells into decellularized ECM hydrogels derived from rat and human breast tissues and build larger volumes of tumor organoids.^122^ Maloney et al.^123^ used an immersion printing technique to print glioblastoma (GBM) in 96‐well plates. Hou et al.^124^ used magnetic bioprinting to consistently produce pancreatic cancer organoids in standard flat‐bottomed 384‐well and 1536‐well plates which may be very useful for scaling up PDOs production and improving the efficiency of drug screening. In addition, their bioprinter significantly increases the formation of breast cancer organoids in 3D collagen gels and precisely generates tumoroid arrays. They further demonstrated the ability of 3D bioprinting to study breast cancer tumorigenesis and microenvironmental control by co‐printing breast cancer cells with normal breast epithelial cells.^125^ In addition, 3D printing can precisely control the shape and size of tumor organoids by controlling the number of cells and their coordinates. Reid et al.^126^ demonstrated that only 10 cells could be used to form 3D structures in a single print. Over time, these structures can fuse with each other to form larger individual organoids, even though the spacing between prints can be as much as 500 μm. Both linear and nonlinear structures can generate dimensions up to 3 mm in length/diameter. These findings suggest that the synergistic application of bioprinting with organoids has great potential in preclinical model development and optimization.

#### 3.2.4 RWV vessel bioreactors

RWV bioreactors are suspension culture systems that were originally developed by NASA for microgravity and Earth‐based cell science experiments.^127^ It simulates microgravity by rotating to create a microgravity, low‐turbulence, low‐shear culture environment for cells, in which cells can grow, self‐aggregate, or aggregate into 3D structures around microcarriers or scaffolds.^128^ RWV bioreactors may improve cell distribution and differentiation, together with nutrient and metabolite transport in cell‐inoculated scaffolds.^129^, ^130^ Moreover, the adverse effects of shear on cells are reduced, including induction of caspase‐mediated apoptosis,^131^ damage to fragile organoid substructures,^132^ and perturbed cellular metabolism.^133^, ^134^ RWV is commonly used in bone tissue engineering with good effects on in vivo bone repair in animal studies where the derived tissue constructs resemble natural bone.^135^

**FIGURE 3:**
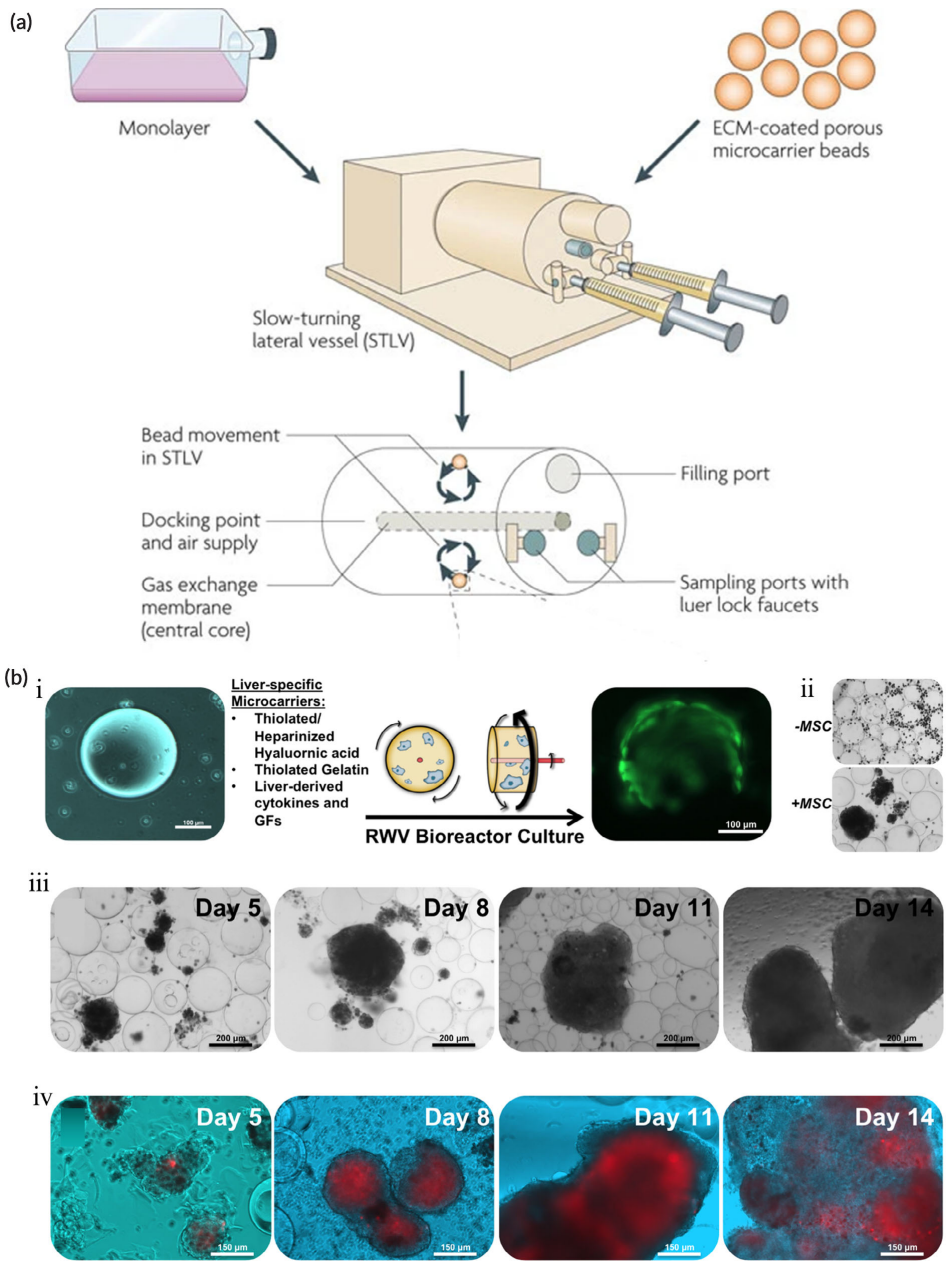
Pattern diagram and structure of cultured tumor organoids in RWV. (a) Operational principles of rotating wall vessel (RWV) technology. (Reproduced with permission from Reference 140. Copyright 2017, IOP Publishing). (b) Colon carcinoma cell growth inside liver tumor organoids. (i) Schematic drawing of liver tumor organoid formation in the RWV bioreactor. (ii) Comparison of organoid cultures with and without MSCs. (iii) Bright light microscopy of liver tumor organoids. (iv) Fluorescence and bright light superimposed image of progressive growth of HCT116 cells within liver tumor organoids. (Reproduced with permission from Reference 142. Copyright 2010, Springer Nature).

In recent years, successful organoids based on RWV bioreactors were developed for the bladder,^136^ vaginal epithelial tissue,^137^ glioblastoma,^138^, ^139^ hepatocellular carcinoma,^140^ and prostate cancer.^141^ Cells cultured in RWV bioreactors resemble their in vivo parental tissues in many respects, including 3D spatial organization and polarity, cell differentiation, function, and response to external stimuli.^142^, ^143^ Skardal et al.^144^ inoculated colon cancer cells (HCT116) in RWV bioreactors cultured with the hepatocyte cell line (Hep‐G2)‐derived liver organoid, wherein tumor cells express mesenchymal and metastatic markers in vivo and exhibit a phenotype similar to colon cancer metastasis in vivo. This tumor organoid is a more suitable model for metastatic tumors than 2D cell lines. Shortly thereafter, the same research team cultured host‐liver colorectal tumor organoids composed of primary human hepatocytes, mesenchymal stem cells (MSC), and colon cancer HCT116 cells in RWV bioreactors to make the tumor organoid model more consistent with in vivo characteristics. The RWV‐based tumor organoids have formed a good physiological correlation with tumor cells in vivo, which well generalizes the responses of tumor and stromal cells to chemotherapeutic agents. Furthermore, the generation of a large number of organoids in a single batch is achievable (Figure 3a).^140^ Overall, the RWV bioreactor operating procedure is relatively simple, flexible, and suitable for different complex models of cell co‐culture and high‐throughput models for drug screening (Figure 3b).^142^, ^145^ However, bubbles sometimes form because of the gas exchange system in RWV bioreactors which leads to increased turbulence and fluid shear stress; this may adversely affect cell aggregation, viability, and differentiation which impairs organoid formation.^146^

#### 3.2.5 Clustered regularly interspaced short palindromic repeats‐Cas9

**FIGURE 4:**
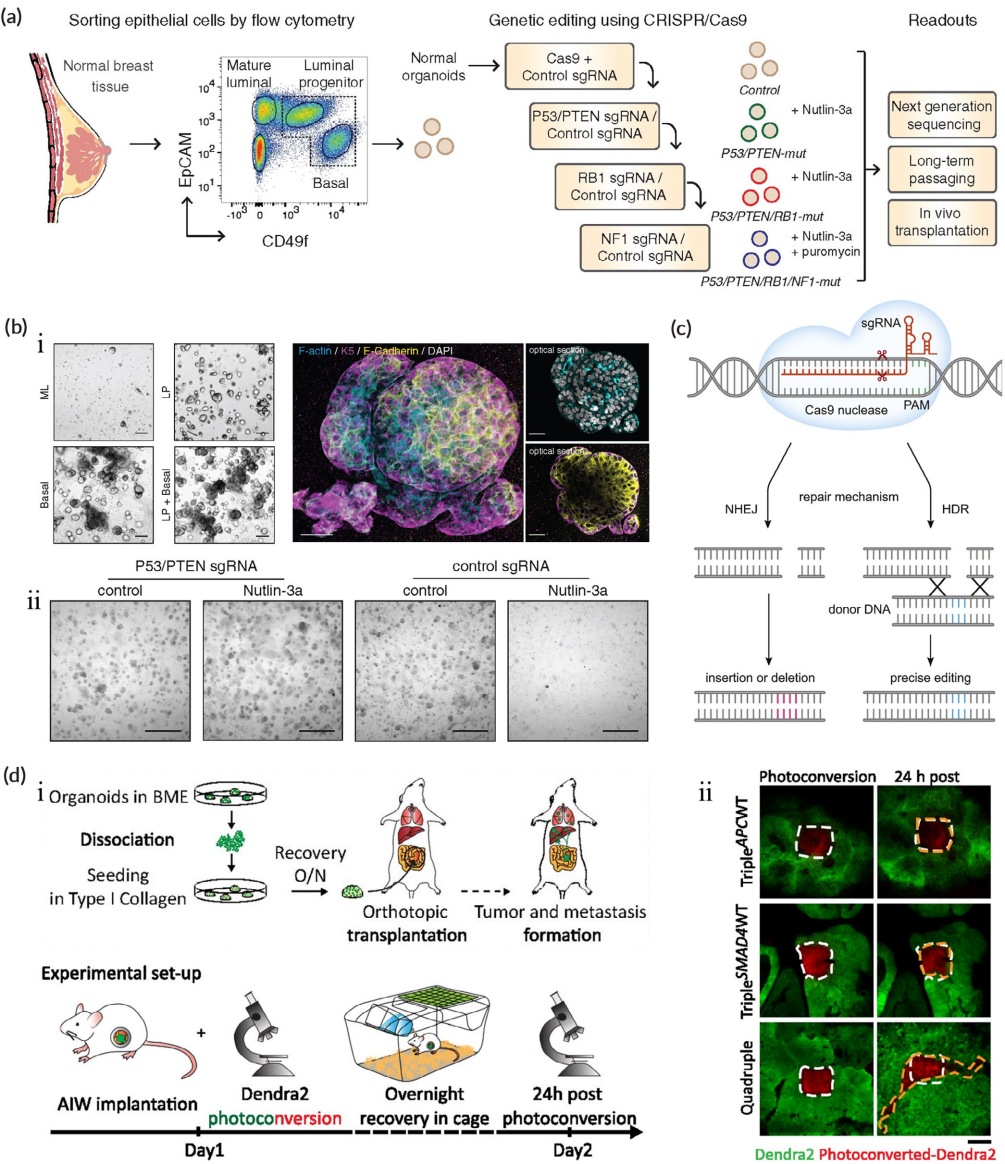
The application of CRISPR‐Cas9 in tumor organoid culture and organoids in the study of cancer invasion and progression. (a) Process of cancer modeling of human breast organoids. (b) Images of gene‐edited human breast organoids. (i) Whole‐mount three‐dimensional confocal image (left) and optical sections (right) of organoids(scale bar 200 μm; scale bar 30 μm). (ii) Representative brightfield images of control organoids or CRISPR‐Cas9‐edited organoids(scale bar 200 μm). (Reproduced with permission from Reference 157. Copyright 2020, Oxford University Press). (c) The mechanism of CRISPR/Cas9. (Reproduced with permission from Reference 155. Copyright 2018, Elsevier Ltd). (d) Invasive behavior of engineered colon cancer organoids after orthotopic transplantation. (i) Flow chart of orthotopic transplantation model and in vivo imaging. (ii) In vivo imaging shows high migratory behavior of tumor cells in colon cancer organoids(scale bar 100 μm). (Reproduced with permission from Reference 176. Copyright 2017, PNAS).

Clustered regularly interspaced short palindromic repeats (CRISPR)‐Cas9 is a genome‐editing tool that targets and modifies specific DNA sequences in the genome.^147^, ^148^ The mechanism by which CRISPR‐Cas9‐mediated genome editing achieves activation/silencing of target sequences of target genes consists of the following three steps: (1) guide RNA targeting to recognize sequences of target genes, (2) Cas9 nuclease upstream of target sequences to break the double strand of the target gene, and (3) ligation or repair of the double‐strand break.^149^ CRISPR‐Cas9 has been rapidly developing since its first application in RNA‐guided DNA cleavage in mammalian cells in 2013. Currently, CRISPR‐Cas9 is widely used to discover new targets for cancer therapy^150^, ^151^ and to identify genes that synergize with‐ or confer drug resistance.^152^, ^153^ Advances and developments in CRISPR‐Cas9 have helped reduce the difficulty of gene editing^154^ which has benefited organoid models (Figure 4c).^155^ It has enabled scientists to obtain tumor organoids from normal tissues, which distinguishes them from previous tumor organoids derived from the patient's tumor tissues. For example, the introduction of multiple mutations from human colorectal tumors into normal human intestinal epithelial organoids by CRISPR‐Cas9 showed that the isogenic organoids carrying these mutations exhibit tumorigenicity in mice.^156^ Similarly, Dekkers et al.^157^ obtained specific subtypes of breast cancer organoids after the targeted knockdown of four breast cancer‐related suppressor genes (P53, PTEN, RB1, and NF1) in normal breast cancer cells using CRISPR‐Cas9 (Figure 4a,b). Meanwhile, tumor organoids established by CRISPR‐Cas9 help determine whether genes are involved in tumorigenesis and their role in tumor development which advances the exploration and study of the mechanisms of carcinogenesis. For example, RBMS3 silencing in benign lung tumor organoids using CRISPR/Cas9 gene editing promotes the growth and progression of malignant lung cancer. Vaishnavi et al.^158^ further analysis shows the role of RBMS3 as a suppressor of lung cancer and the possibility that RBMS3 silencing could lead to malignant progression. Notably, the newly developed CRISPR‐Cas9‐mediated homology‐independent organoid transgenesis (CRISPR‐HOT) system exhibits rapid and efficient generation of genetically engineered human liver duct organoids and human fetal hepatocyte organoids which have promising applications in cancer modeling and gene function studies.^28^ However, the current CRISPR‐Cas9 technology is imperfect. Guide RNAs show poor function in organoids compared to cell lines, possibly because the existing methods do not accurately predict active gRNAs.^159^ In addition, CRISPR‐Cas9‐mediated genome editing has the following limitations that need to be addressed: off‐target effects, original spacer adjacent motif requirement, DNA‐damage toxicity, and immunotoxicity.

#### 3.2.6 Air–liquid interphase

Traditional organoid culture methods cannot meet the requirements of in vitro simulation of TME, so researchers developed air–liquid interphase (ALI), a co‐culture of tumor tissue/cells with TME component cells. ALI is primarily achieved by placing tumor cells or chopped tumor tissue, endogenous homologous tumor‐infiltrating lymphocytes (TILs), and pre‐cured collagen gels containing Transwell into culture wells spiked with medium, exposing the organoid to air on one side and liquid medium on the other, a structure that facilitates increased oxygen concentration in the culture system.^160^ ALI PDO encapsulates the T‐cell receptor spectrum of primordial origin and can functionally mimic PD‐1‐dependent immune checkpoint blockade, causing TILs activation and expansion leading to further cytotoxic responses, and in which immune cells can be retained for 30 days.^161^ Compared to the traditional culture approach of PDO, ALI PDO can achieve a more accurate overview of the stem cell niche and mimic tumor cell–stromal cell interactions by co‐culturing tumor cells with stromal components, allowing studies to focus more on the intra‐tumor immune response.^50^

### 3.3 Analysis and characterization of PDO

The established PDO model needs to be identified and characterized before proceeding to the next step of the experiment. PDO is most widely used as a model for drug evaluation and screening. Cell viability assays, on the other hand, are the main method to measure the response of the organoid to the tested drug, such as the CellTiter‐Glo® luminescent cell viability assay (Promega).^97^ In addition to functional analysis, genome‐wide, and targeted CRISPR screens, single‐cell transcriptomics, image‐/imaging‐based assessment methods.^162^ The combined use of multiple analytical methods (e.g., cell viability assays, single‐cell sequencing, histopathology, and real‐time imaging combined) can improve the predictive accuracy of organoids as drug screening models to some extent.^163^ High content screening (HCS) uses imaging, image analysis, and data extraction to achieve multiple phenotypic data in organoids HCS uses imaging, image analysis, and data extraction to obtain multiple phenotypic data from a single cell in the organoid. Compared to single‐endpoint measurements of high‐throughput screening, the multiparametric readouts of HCS enable it to reveal multiple phenotypic changes and intercellular differences in cells in response to PDO drugs.^164^

## 4 APPLICATION OF TUMOR ORGANOIDS

### 4.1 Study of tumorigenesis and development mechanisms

Tumorigenesis and progression are based on the accumulation of genetic mutations. Determining the mutational process is important for a deeper understanding of tumors. The introduction of pathological mutations into normal organoids by genetic modification to mimic the tumorigenic process is feasible.^156^, ^157^, ^165^, ^166^, ^167^, ^168^, ^169^, ^170^ Matano et al.^156^ introduced driver pathway mutations (APC, SMAD4, TP53, KRAS, and PIK3CA) using CRISPR‐Cas9 genome‐editing technology, and established selective culture conditions in normal human colonic organoids to allow multiple mutation combinations in isogenic organoids. This class of organs exhibits tumorigenic capacity proportional to the number of mutations it carries after implantation in mice under the kidney subcapsule. This mouse model exhibits tumorigenicity compared to CRC organoids after the insertion of oncogenic mutations in human adenoma organoids with the chromosomal instability (CIN) phenotype.^156^ Similarly, mutant organoids with invasive carcinoma features grow after transplantation into mice as tumors with the combined loss of APC and TP53 leading to the appearance of extensive aneuploidy (a hallmark of cancer progression).^165^ In contrast, tumor cells show significantly higher mutation rates, and most of the mutations were acquired during the final dominant clonal expansion of cancer when comparing genetic differences between normal and tumor organoids using whole‐genome sequencing.^166^, ^167^ In addition, the mesenchymal niche of mutant stem cells is associated with tumorigenesis, and a paracrine coordinating mechanism is identified in organoid models.^168^

Metastatic progression is a major cause of death in cancer patients and the migration and invasion of cancer cells are key drivers of cancer metastasis.^171^ Co‐culture of near‐physiological microvessels with breast cancer organoids shows that tumor cells rapidly remodel, destroy, or integrate into existing vessels and form mosaic vessels which allow tumor cells to disseminate.^172^ Meanwhile, Homophilic CD44 interactions between tumor cells and subsequent CD44–PAK2 interactions mediate migration and aggregation of circulating tumor clusters in breast cancer organoids, with aggregated tumor cells promoting destructive metastasis of tumors.^173^ Furthermore, basal epithelial gene expression triggers collective invasion in major human breast cancer subtype organoids.^174^ Similarly, inhibition of ROCK2 activity increases the invasiveness of colorectal adenocarcinoma organoids.^175^ Clonal selection and metastatic capacity exhibit ecotype dependence (Figure 4d),^176^, ^177^ but this stem cell niche factor dependence is progressively lost with cancer progression.^178^

### 4.2 Drug development and screening

**FIGURE 5:**
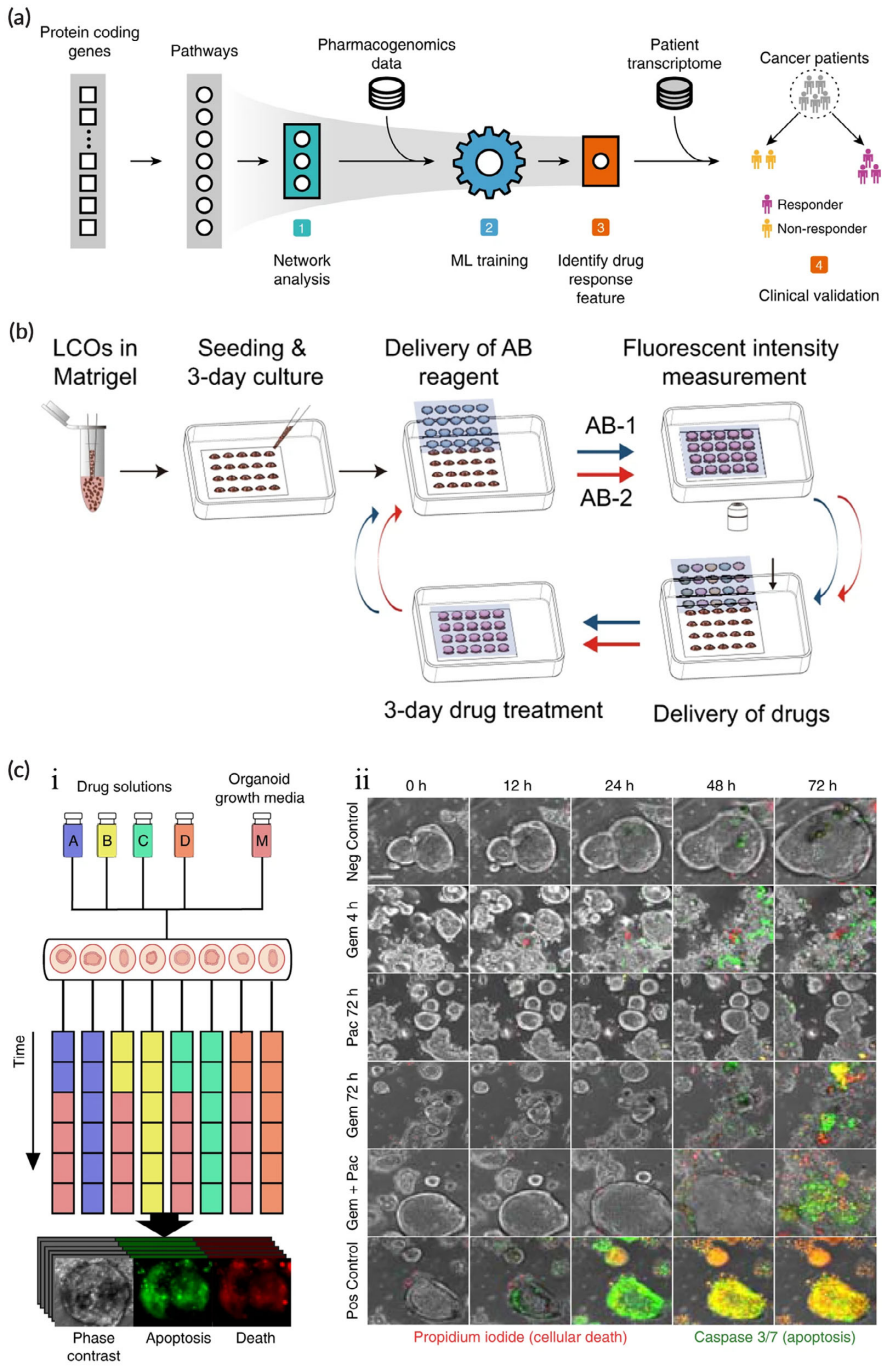
Application of tumor organoids in drug screening. (a) Process for organic identification of drug response biomarkers by network‐based machine learning (ML). (Reproduced with permission from Reference 185. Copyright 2020, Springer Nature). (b) Flow of a week‐long drug sensitivity test on the InSMAR‐chip. (Reproduced with permission from Reference 181. Copyright 2021, Springer Nature). (c) Combinatorial drug treatment of human tumor organoids on microfluidic platform. (i) continuous fluorescence and phase imaging of drug treatment and stimulation during treatment. (ii) Different drug and concentration combinations for treatment of organoid and normal growth media(scale bar 100 μm). (Reproduced with permission from Reference 85. Copyright 2020, Springer Nature).

Statistics from 2010 to 2017 show that the success rate of drug candidates from phase I clinical trials to the initiation of late‐stage development is below 10%, and the main reason for their failure is inadequate safety or efficacy.^179^ Therefore, the establishment of effective preclinical models is beneficial to improve the success rate of drug development. 2D cell lines, PDXs, and PDOs can all be used as preclinical models for drug screening of tumors. Numerous studies show that PDOs pathologically characterizes and maintains genetic stability and can more accurately assess drug efficacy and drug sensitivity than 2D cell lines and PDXs (Figure 5b).^180^, ^181^ Moreover, uniform‐sized organoids can be obtained by using simple microfluidic devices for repeatable drug screening.^182^ Grossman et al.^183^ found that drug sensitivity test results for pancreatic ductal adenocarcinoma (PDAC) PDOs correlate with the clinical response of individual patients. The use of drug sensitivity data excludes ineffective drugs for patients and reduces the toxic reactions produced by these drugs. A study of 27 primary liver cancer organoid lines from different regions of the tumor (from 129 patients) shows that some anti‐cancer drugs are broadly effective against multiple lines, while others were effective against only one organoid class.^184^ Furthermore, PDOs can provide preclinical models for therapeutic studies of rare tumors. Puca et al.^49^ established a tumor organoid for neuroendocrine prostate cancer, and a fraction of single drugs and drug combinations in high‐throughput drug screens show new possibilities regarding the treatment of this disease. Moreover, the application of automated microfluidics to drug screening of tumor carcinoids allows the real‐time analysis of carcinoids. Chronological administration of drug treatments might be more effective than constant doses of individual drugs or combinations (Figure 5c).^85^ Kong et al.^185^ used pharmacogenomic data of colorectal and bladder carcinoids for network‐based machine learning, and the identified biomarkers accurately predict drug responses in 191 clinical patients (Figure 5a).

The inadequate safety of drug candidates is another major reason for drug development failure, and matching tumor organoids to normal organoids has potential applications in estimating drug safety and appropriate dosing. Herpers et al.^186^ produced more than 500 dual‐targeting bispecific antibodies (bAbs) targeting the Wingless‐related integration site (WNT) and receptor tyrosine kinase (RTK). A large‐scale screening of bAbs was performed using a heterogeneous colorectal cancer PDOs biobank matched to normal colonic mucosal samples. The results identified that MCLA‐158 shows therapeutic benefits to colorectal cancer cells and is minimally toxic to normal LGR5+ colon stem cells. Photodynamic therapy (PDT) is an anti‐tumor therapy that relies on photosensitizers (PS), light activation at specific wavelengths, and oxygen levels to achieve tumor targeting through rapid accumulation of PS at the tumor site and light excitation of PS to produce reactive oxygen species. The 3D structure of the PDO model allows the cells in it to be in different oxygen concentration environments, which facilitates the testing of PDT efficacy.^187^ Nanobody‐targeted PDT remains therapeutic in tumor head and neck squamous cell carcinoma (HNSCC) organoids with low EGFR expression levels, whereas the corresponding normal organoids did not respond to EGFR‐targeted PDT.^188^ Valančiūtė et al.^189^ tested the killing effect of methylene blue photodynamic therapy (MB‐PDT) in combination with light emitting diodes (LEDs) using a lung cancer carcinoid model. The results showed that MB‐PDT destroyed the structure of lung carcinoids and caused tumor cell death by inducing immunogenic cancer cell death (ICD). This suggests that the organoid could be an ideal platform for testing and evaluating PDT, providing more valuable data for preclinical studies of PDT.

Tumor organoids usually retain the genetic characteristics and heterogeneity of parental tumors, which offers the possibility to investigate the mechanisms of drug resistance and find new therapeutic targets.^45^ CD44+ hepatocellular carcinoma (HCC) PDOs is significantly resistant to sorafenib, and sorafenib increases Hedgehog signaling protein and CD44 levels.^190^ Meanwhile, sorafenib resistance is reversed in CD44+ HCC PDOs following the introduction of a Hedgehog signaling inhibitor (GANT61).^190^ Stromal antigen (STAG) 3 promotes colorectal cancer cell migration and chemoresistance suggesting that STAG3 may be a potential therapeutic target and prognostic biomarker for colorectal cancer.^191^ Similarly, microRNA 21 (miRNA21) may mediate resistance to HSP90 inhibitors in cholangiocarcinoma (CCA) organoids by reducing DNAJB5 levels. In other words, HSP90 inhibitors potentially treat CCA, while the presence of miRNA21 may reduce its effectiveness in treatment; therefore, its levels could be used as a diagnostic marker for suitability of the treatment method.^192^

Although organoids have made significant contributions to the field of drug screening, the influence of their structure, spatial distribution, and size on drug efficacy should not be ignored. Studies have shown that compared with individual organoid cells, 3D organoids are less sensitive to drugs, and their efficacy is significantly lower. The presence of stents can delay the effective action time of drugs, and Matrigel can cause more obvious delays than synthetic hydrogels. In addition, among larger organoids (with a cross‐sectional area > 8000 μm^2^), the closer the cells were to the core, the later the drug response occurred. No similar phenomenon was observed in small organoids.^193^ Hence, organoid‐based drug sensitivity data warrant further research and investigation by taking various factors into account.

### 4.3 Precision medicine

Precision medicine (also known as personalized medicine) involves the analysis of genetic traits and differences between individuals through genomics and uses the results to guide the development of effective interventions. Clinical, environmental, behavioral, and lifestyle aspects also need to be considered in this process.^194^, ^195^ Precision medicine requires preclinical models to closely resemble the in vivo environment to develop treatment plans and predict the clinical response to therapy. Organoids have potential applications in precision medicine research because of their good generalization of the in vivo environment.^196^, ^197^

**FIGURE 6:**
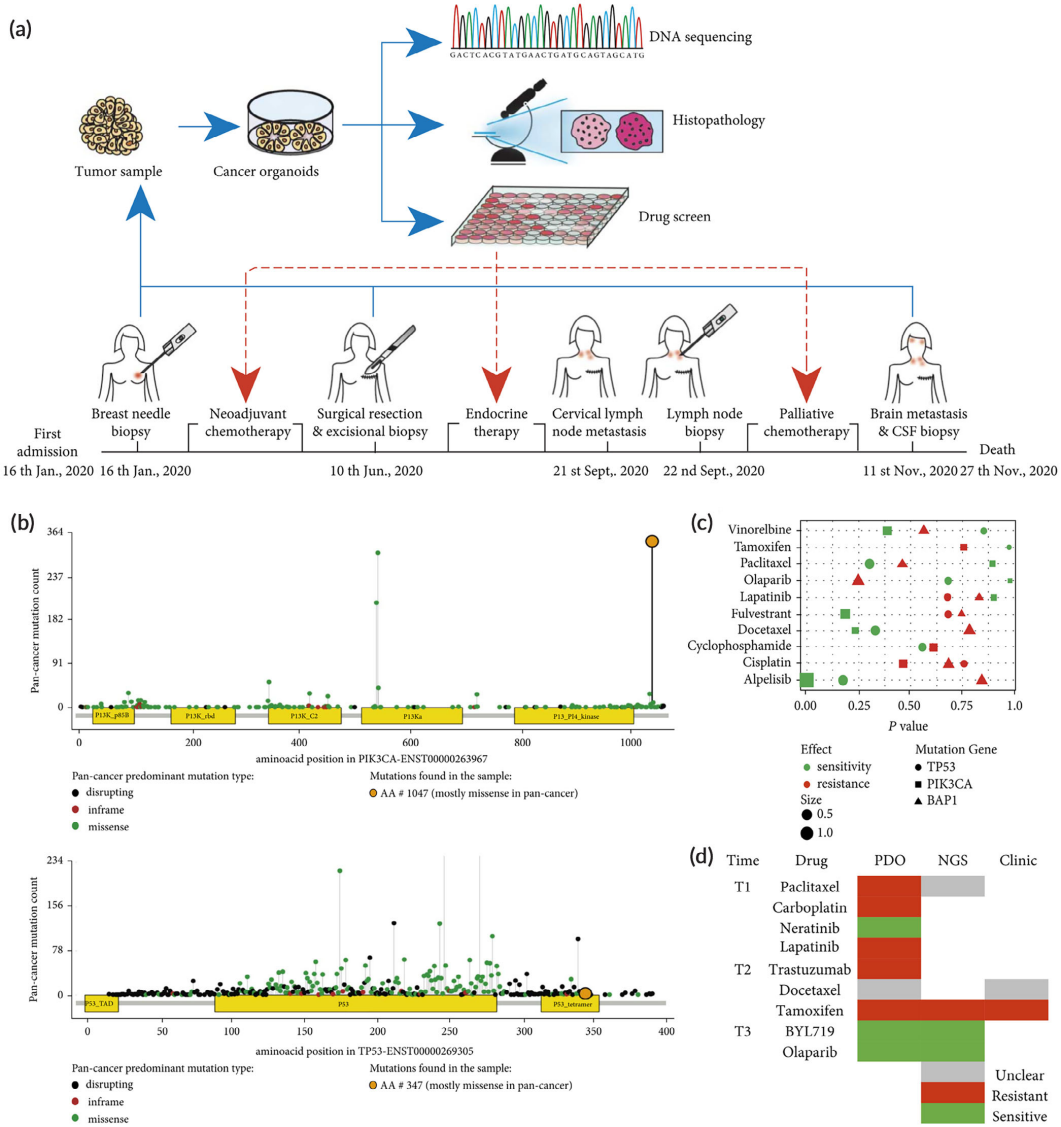
The application of breast cancer organoids in precision medicine. (a) Personalized treatment of metastatic ER+ breast cancer in a 41‐year‐old woman using tumor organoids. (b) Patients' mutations in PIK3CA and TP53 genes. (c) Scatter plot of the impact of drug treatment associated with gene mutations in the GDSC database. (d) Drug response prediction for PDOs and NGS and clinical response. (Reproduced with permission from Reference 202. Copyright 2022, Y. Liu et al.).

Tumor organoids are known as precision oncology when applied to precision medicine.^198^ Precision oncology provides an overview of the genetic and histological characteristics of a patient's tumor, inter‐, and intra‐tumor heterogeneity, and drug response and resistance. It also allows drug screening and safety testing to determine which patients are most likely to benefit from it and to develop an appropriate treatment plan (Figure 6a–d).^199^, ^200^, ^201^, ^202^, ^203^ A proven treatment regimen will help increase patient survival and improve quality of life.^204^ Vlachogiannis et al.^200^ established a living biobank for PDOs derived from patients with metastatic gastrointestinal cancer. The phenotype and genotype of PDOs are similar to those of the parental tumor and molecular profiling is consistent with the drug screening results. The PDOs have 88% positive predictive value and 100% negative predictive value in forecasting patient response to targeted therapy or chemotherapy. Pauli et al.^199^ combined genomic sequencing data with high‐throughput drug screening of tumor organoid models to develop a series of optimal treatment regimens. A combination of the PIK3 inhibitor (buparlisib) with the hypoxia signaling inhibitor (vorinostat) was identified as the optimal treatment option for patients with uterine carcinosarcoma. Meanwhile, the combination of buparlisib with PARP and HDAC inhibitor Olaparib was identified as the optimal treatment regimen for patients with endometrial adenocarcinoma, and there was good concordance between in vitro and in vivo tumor responses. Seppälä et al.^201^ found that next‐generation sequencing (NGS) after biomass expansion of pancreatic ductal adenocarcinoma organoids improves detection of somatic mutations and quantifies copy number variants which improves clinical sequencing quality. Moreover, Song et al.^203^ established 15 monoclonal tumor organoid lines from four different lesions in one colon cancer patient and each PDOs line showed heterogeneity in genotype and phenotype. This revealed time‐point heterogeneity in anticancer drug responses that can be used to pinpoint the optimal time frame for therapeutic drugs combined with high‐throughput drug screening and image‐based drug responsiveness tracing. The application of organoids significantly shortens the time to derive the analytical results used to guide treatment and facilitate real‐time patient assessment compared with traditional precision medicine research methods.^205^ Notably, the association between predicted and true patient outcomes requires further validation in large‐sample studies.

### 4.4 Immunotherapy

**FIGURE 7:**
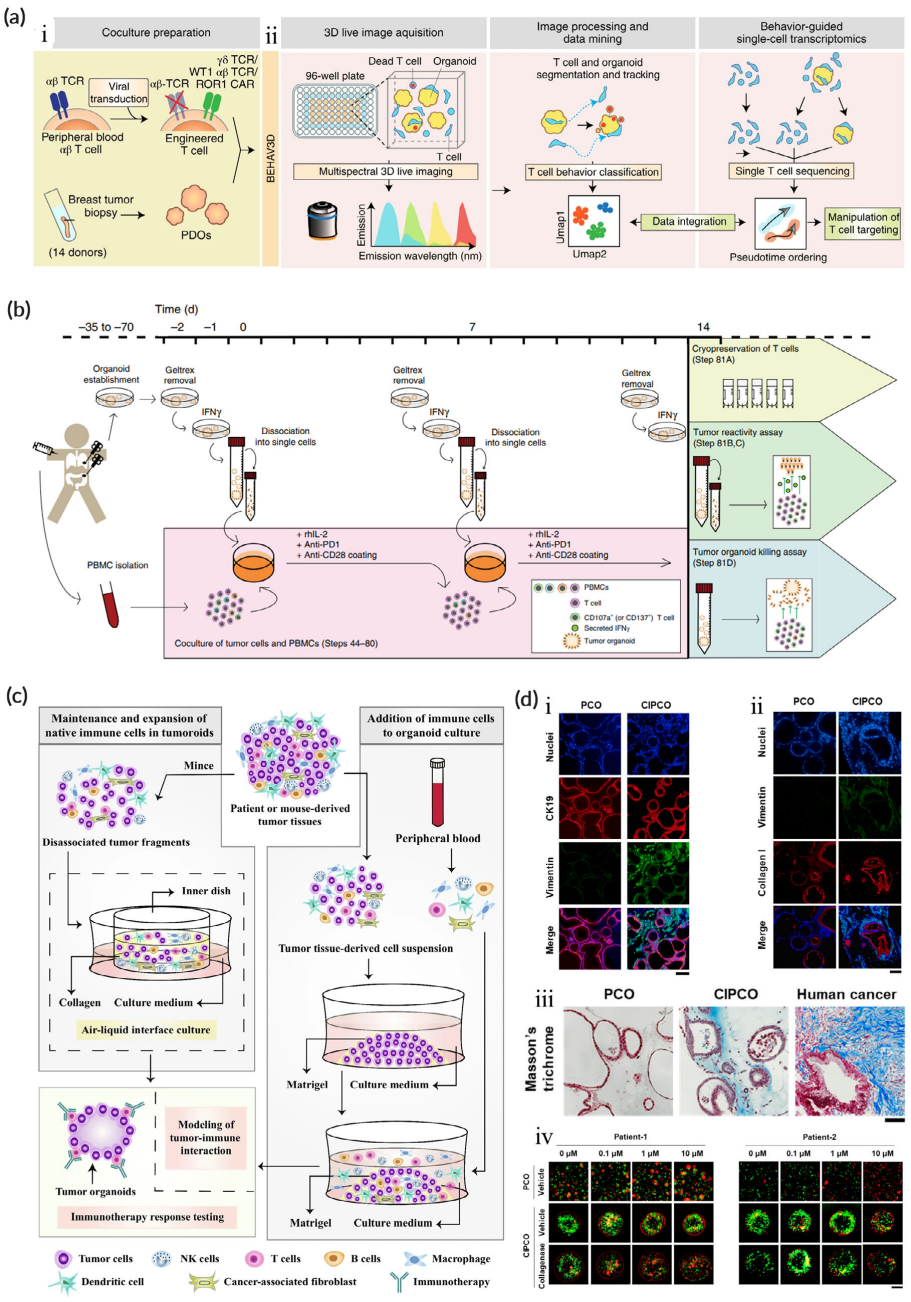
Tumor organoids are used as in vitro models for immunotherapy and simulation of tumor microenvironment in tumor organoids. (a) Flow of TEG co‐culture with PDOs. (i) Schematic diagram of TEG co‐culture with PDOs. (ii) BEHAV3D platform. (Reproduced with permission from Reference 210. Copyright 2023, J. F. Dekkers et al.). (b) Schematic diagram of PBMC co‐culture with patient‐derived tumor organoids. (Reproduced with permission from Reference 212. Copyright 2019, Springer Nature). (c) Schematic diagram of tumor organoids and immune cells co‐cultured to mimic the microenvironment. (Reproduced with permission from Reference 225. Copyright 2022, Elsevier Inc). (d) Morphological features of cancer‐associated fibroblast‐integrated pancreatic cancer carcinoid organs (CIPCO). (i) Immunofluorescence images of CK19‐ and vimentin‐labeled PCO and CIPCO models (scale bar 100 μm). (ii) Immunofluorescence images of type I collagen‐labeled PCOs and CIPCOs (scale bar 100 μm). (iii) Comparison of Masson's trichrome‐stained images of PCOs, CIPCOs, and human pancreatic cancer cells (scale bar 500 μm). (iv) Immunofluorescence detection of the sensitivity of cancer cells to different concentrations of gemcitabine in PCO and CIPCO models (scale bar 200 μm). (Reproduced with permission from Reference 226. Copyright 2022, Y.‐H. Go).

Immunotherapy is a new anti‐tumor strategy that uses the patient's immune system to kill tumor cells. Tumor‐specific mutations generate neoantigens and the immune system triggers a sufficient immune response to specifically kill them when the tumor cell exhibits sufficient immunogenicity.^206^, ^207^ Therefore, we can choose to actively target specific antigens on the tumor or augment the host immune system, including monoclonal antibodies, adoptive transfer of TILs, allogeneic cell‐based vaccines, chimeric antigen receptors T (CAR‐T) cells, and immune checkpoint inhibition when the strength of the immune response induced by tumor cell neoantigens is insufficient.^208^, ^209^ Currently, organoid models are used in tumor immunology studies including exploring potential immune mechanisms,^210^, ^211^ immunotherapy modeling,^161^, ^212^, ^213^ and personalized immunotherapy testing.^214^, ^215^, ^216^ T cells are key players in immunotherapy, and the co‐culture of autologous tumor organoids and peripheral blood lymphocytes could be used from mismatch repair‐deficient colorectal cancer and peripheral blood from patients with nonsmall cell lung cancer to enrich tumor‐reactive T cells and assess the efficiency of T‐cell‐mediated killing of matched tumor organoids.^217^ This co‐culture model can also be used to assess the sensitivity of patients to immunotherapy and explain the possible mechanisms of drug resistance (Figure 7b).^212^

The TME can affect the immune system and immunotherapy, and understanding the interactions between the two can help optimize immunotherapy. Neal et al.^161^ established primary tumor epithelial and endogenous immune matrix organoids in humans and mice using ALI approach and showed that tumor organoids in TILs encapsulate the primitive tumor T‐cell receptor (TCR) profile. Also, tumor organoids can mimic immune checkpoint blockade (ICB), anti‐PD‐1‐ and/or anti‐PD‐L1 amplification, activation of tumor antigen‐specific TILs, and trigger tumor cytotoxicity. In addition, microfluidic platforms allow tumor organoids to be co‐cultured with TME components which provides models for studying immune mechanisms in the TME, screening immunotherapeutic agents, and predicting clinical responses to therapy.^218^

CAR‐T cells therapy is to recognize and attack tumor cells by modifying autologous or allogeneic T cells to target specific antigens of tumor cells. Currently, CAR‐T cells are mainly used in hematologic cancers and not widely used in solid tumors, but related research is rapidly developing. There is a possibility of adverse reactions during CAR‐T cells therapy, such as cytokine storm, and neurological toxicity, which may even threaten life safety.^219^ Therefore, preclinical evaluation of the efficacy and toxicity of CAR‐T cells is necessary. As a preclinical model of CAR‐T cells, the advantages of PDO in retaining parental tumor cell heterogeneity and specific antibody expression give it a unique advantage. A live confocal imaging protocol was established that allows monitoring of effector cell recruitment and cytolytic activity at the level of individual colorectal carcinoids to assess CAR efficacy and cytotoxicity.^214^ Jacob et al.^220^ co‐cultured EGFRvIII‐specific CAR‐T cells with EGFRvIII+ and EGFRvIII‐ GBO, respectively, and proliferation of CAR‐T cells and a decrease in EGFRvIII/EGFR signaling intensity were clearly observed in EGFRvIII+ GBO. Further experiments revealed that the T‐cell killing effector granzyme B in the co‐culture model of CAR‐T cells with EGFRvIII+ GBO was clustered on the side close to the tumor cells expressing EGFRvIII, and the levels of cytokines suggestive of antigen recognition and T‐cell activation were also significantly increased.

Moreover, Teijeira et al.^215^ co‐cultured tumor organoids, autologous fibroblasts, and T cells to test the therapeutic response of CEA‐CD3 T‐cell engagers against colon cancer and test drug combinations. Recently, Dekkers et al.^210^ described a system that characterizes the behavioral phenotypic heterogeneity of cellular immunotherapies and investigates the dynamic interactions between immune cells and tumor organoids through imaging and transcriptomics (Figure 7a). These findings suggest that organoids have promising applications in tumor immunotherapy.

### 4.5 Simulation of the TME

The TME is a hypoxic and acidic environment produced by the tumor and includes cellular components such as tumor‐associated fibroblasts CAFs, endothelial cells, bone marrow‐derived suppressor cells (MDSC), immune cells, and noncellular components such as the ECM, cytokines, and growth factors.^221^ The TME is shaped and governed by the tumor and plays an important role in tumor growth, progression, invasion, and acquisition of drug resistance. Its dynamic regulation leads to suppression of the body's immune cell function or apoptosis of anti‐tumor effector cells; thus, the tumor acquires an immune escape capability which is the main reason for immunotherapy failure.^222^ Fortunately, the stromal cell types within the TME are genetically stable which represents the possibility of reducing tumor recurrence and drug resistance by targeting the TME.^223^

The application of tumor organoids in the simulation of TME is reported (Figure 7c,d).^224^, ^225^, ^226^ Tsai et al. co‐cultured human pancreatic cancer organoids, CAF, and T cells to establish an organic co‐culture model and observed ECM infiltration around tumor tissue which may be valuable for the evaluation of immunotherapeutic agents in the context of T‐cell infiltration.^227^ Neal et al.^161^ combined tumor organoids, CAFs, and TILs using the ALI method in co‐culture to preserve endogenous immune and nonimmune matrix elements and the in vivo association and function between native TILs and tumor cells. This allows for immuno‐oncology studies and personalized immunotherapy testing. However, fibroblasts and immune cells in these PDOs are progressively reduced and can only be maintained for 1–2 months, suggesting that they can only be used for short‐term disease modeling.^161^ CAFs play an important role in cancer because they secrete various ECM components. They promote the growth of tumor organoids through paracrine signaling and make tumor organoids resistant to anticancer drugs to create a favorable environment for tumor development.^228^ Öhlund et al.^229^ co‐cultured mouse pancreatic stellate cells (PSCs) and pancreatic ductal adenocarcinoma (PDA) organoids. PSCs co‐cultured with tumor organoids acquired a CAF phenotype and promoted organoid proliferation compared to naive PSCs embedded in Matrigel alone. Furthermore, they described two PSC‐derived coexisting CAF isoforms with conflicting functions showing myofibroblastic and inflammatory phenotypes. Further research suggests that IL1 induces LIF expression and downstream JAK/STAT activation to generate inflammatory CAFs, whereas TGFβ antagonizes this process by downregulating IL1R1 expression and promoting differentiation into myofibroblasts.^230^ The heterogeneity of CAFs within tumors suggests that selective targeting of CAFs may be beneficial for anti‐tumor therapy.

## 5 CONCLUSIONS AND PROSPECTS

Malignant tumors are one of the leading causes of death and impose an increasingly heavy burden on every country. Therefore, it is important to further understand the mechanisms of tumorigenesis and tumor development, develop safer and more effective drugs, and formulate more personalized treatment plans. The establishment of research models that closely resemble the original tumor characteristics will help solve these challenges. Previous tumor studies have relied on 2D cell line models and PDXs models; however, both have non‐negligible shortcomings. Tumor organoids provide a better simulation of the histological, phenotypic, and genotypic characteristics of parental tumors and retain inter‐ and intra‐tumor heterogeneity compared with 2D cell lines and PDXs models while avoiding animal‐related ethical issues. Meanwhile, the synergistic combination of tumor organoids with emerging technologies such as tissue engineering scaffolds, microfluidics, 3D bioprinting, bioreactors, and gene editing overcome some of the drawbacks of traditional culture methods and broaden the application of tumor organoids. In addition, the establishment of several types of tumor organoids, such as colorectal cancer, liver cancer, and breast cancer organoids, provide valuable in vitro models for the study of the mechanisms of the corresponding tumors, drug development and screening, and optimization of precision medicine and immunotherapy. In recent years, the development of iPSC‐ and circulating tumor cells(CTC)‐derived organoids has enriched the sources of tumor organoids and contributed to personalized medical treatment of non‐surgical tumor patients.^231^ Moreover, iPSC‐derived organoids can retain tumor and nontumor cell components which helps restore cell–cell interactions within the tumor.^232^

Tumor organoids provide models for tumors at various stages of development by combining genetic modifications and various omics analyses from the establishment of organoids to the creation of living biobanks through cryopreservation. However, they only partially simulate the characteristics and development of tumors. The following limitations persist: (1) the lack of standardized culture protocols and evaluation criteria and the low success rate of some tumor organoids prevents the reproducibility and repeatability of organoids and is not conducive to high‐throughput assays. Therefore, the development of standard culture and evaluation protocols for tumor organoids and the use of well‐defined materials are needed to improve the generation efficiency and provide more reliable results for tumor organoids as in vitro models. (2) Tumor organoids are mainly derived from epithelial cells and the number of studies on tumor organoids derived from nonepithelial cells is limited. Further focus on the establishment of nonepithelial‐derived tumor organoids will be beneficial to mechanistic studies and therapeutic optimization of related tumors. (3) Normal cells have an advantage over tumor cells during the long‐term culture of tumor organoids due to their genetic stability^40^ and epigenetic drift may occur in long‐term cultured and passaged tumor organoids.^159^ Therefore, it is necessary to explore the mechanism by which genetic drift occurs and to avoid normal cell contamination to make tumor organoids more mature and stable; some studies have been conducted to overcome this limitation.^233^ (4) Tumor organoids must be enhanced to simulate interactions between cells, tissues, and organs. Although it is possible to co‐culture stromal cells and the ECM to mimic the TME, the role of the peripheral immune system in tumors cannot be assessed^234^ and these models cannot be cultured for long periods of time.^161^ Applying techniques from other models and tissue engineering to tumor organoids may allow for long‐term stable co‐culture of multiple tissues/organs which further improves the accuracy of predicting clinical responses.^235^ Despite the deficiencies of tumor organoids, the combined multi‐organ and multi‐technology approach has great potential to help bridge the gap between tumor organoids and the original tumor to simulate a more complex and realistic state in vivo.

## AUTHOR CONTRIBUTIONS

**Qian Yang:** Visualization (equal); writing – original draft (lead); writing – review and editing (equal). **Mengmeng Li:** Investigation (equal); supervision (equal); visualization (equal). **Xinming Yang:** Methodology (equal); project administration (equal); resources (equal); supervision (equal). **Zian Xiao:** Methodology (equal); project administration (equal); resources (equal). **Xinying Tong:** Data curation (equal); investigation (equal); validation (equal). **Ayinuer Tuerdi:** Data curation (equal); investigation (equal); validation (equal). **Shisheng Li:** Conceptualization (equal); funding acquisition (lead); writing – review and editing (equal). **Lanjie Lei:** Conceptualization (equal); methodology (equal); writing – review and editing (equal).

## CONFLICT OF INTEREST STATEMENT

The authors declare that they have no known competing financial interests or personal relationships that could have appeared to influence the work reported in this paper.

## Data Availability

Data sharing is not applicable to this article as no new data were created or analyzed in this study.
